# Injectable and Conductive Granular Hydrogels for 3D Printing and Electroactive Tissue Support

**DOI:** 10.1002/advs.201901229

**Published:** 2019-08-21

**Authors:** Mikyung Shin, Kwang Hoon Song, Justin C. Burrell, D. Kacy Cullen, Jason A. Burdick

**Affiliations:** ^1^ Department of Bioengineering University of Pennsylvania Philadelphia PA 19104 USA; ^2^ Department of Neurosurgery Perelman School of Medicine University of Pennsylvania Philadelphia PA 19104 USA; ^3^ Center for Neurotrauma Neurodegeneration and Restoration Corporal Michael J. Crescenz Veterans Affairs Medical Center Philadelphia PA 19104 USA

**Keywords:** conductive hydrogels, gallol, injectable hydrogels, metal reduction, microgels

## Abstract

Conductive hydrogels are attractive to mimic electrophysiological environments of biological tissues and toward therapeutic applications. Injectable and conductive hydrogels are of particular interest for applications in 3D printing or for direct injection into tissues; however, current approaches to add conductivity to hydrogels are insufficient, leading to poor gelation, brittle properties, or insufficient conductivity. Here, an approach is developed using the jamming of microgels to form injectable granular hydrogels, where i) hydrogel microparticles (i.e., microgels) are formed with water‐in‐oil emulsions on microfluidics, ii) microgels are modified via an in situ metal reduction process, and iii) the microgels are jammed into a solid, permitting easy extrusion from a syringe. Due to the presence of metal nanoparticles at the jammed interface with high surface area in this unique design, the granular hydrogels have greater conductivity than non‐particle (i.e., bulk) hydrogels treated similarly or granular hydrogels either without metal nanoparticles or containing encapsulated nanoparticles. The conductivity of the granular hydrogels is easily modified through mixing conductive and non‐conductive microgels during fabrication and they can be applied to the 3D printing of lattices and to bridge muscle defects. The versatility of this conductive granular hydrogel will permit numerous applications where conductive materials are needed.

## Graphical abstract

**Injectable and conductive granular hydrogels** are introduced through the jamming of microgels containing gallol redox chemistry inspired by fruit browning phenomena, coupled with an in situ metal reduction process. The conductive hydrogels provide facile 3D printability with desirable conductivity of extruded filaments, as well as electrical tissue conduction for further biomedical applications related to electroactive tissues.

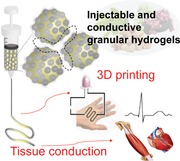

Conductive biomaterials are important to mimic electrophysiological characteristics of tissues, such as neural, skeletal muscle, and cardiovascular tissues, toward applications in tissue repair^1^ and as biosensors,^2^ bioelectrodes,^3^ flexible/wearable electronics,^4^ and electrically controlled drug delivery systems.^5^ There are numerous well‐known conductive polymers, such as polypyrroles,^6^ polyanilines,^7^ polythiophenes,^8^ and poly(3,4‐ethylene dioxythiophenes),^9^ which have been explored as electroresponsive materials and to alter cellular behaviors such as cell differentiation and proliferation.[qv: 7,8a,9b] Metal nanoparticles (e.g., gold and silver nanoparticles) and carbon‐based materials (e.g., carbon nanotubes and graphene) have also been widely explored as additives to alter biomaterial conductivity.^10^ Although conductive materials have been developed for numerous applications, some characteristics such as limited aqueous solubility and brittle mechanical properties may limit their widespread utility.

To present a 3D hydrated network with mechanical properties and conductivity similar to that of biological tissues, electroconductive hydrogels are a promising class of biomaterials.^11^ Often, the aforementioned electroactive materials are simply encapsulated into hydrogels to impart conductivity;^12^ however, a high packing density of the conductive materials is needed for electrical conductivity, resulting in high costs for preparation, poor hydrogel gelation, and altered mechanical properties. Approaches to overcome these limitations have included freeze‐drying the hydrogel to more densely pack the encapsulated materials^13^ or post‐coating of hydrogels after gelation with conductive materials;^14^ yet, these methods do not allow the hydrogels to be injectable, which is important for direct introduction into tissues or in fabrication approaches such as with 3D printing.

Here, we introduce a new concept in the development of injectable and conductive hydrogels, based on the processing of hyaluronic acid (HA) into hydrogel microparticles (i.e., microgels) and their assembly into granular solids that include metal–phenolic coordination^15^ networks (**Figure** **1**). For electrical conductivity, in situ metal reduction is introduced through the inclusion of gallol moieties, which are ubiquitous polyphenols widely found in a variety of plants, fruits, vegetables, and nuts.^16^ The gallol moiety has benzene‐1,2,3‐triols, capable of being oxidized to form galloquinones and to donate two electrons per one molecule. When coupled with this oxidation of gallol, metal ions (e.g., M^+^) are reduced to generate metal nanoparticles (e.g., M^0^). Furthermore, gallols may act as chelators to form coordinated networks with metal nanoparticles. The in situ synthesis of conductive materials from their precursors is an attractive approach when compared to embedding techniques to improve conductivity and mechanical properties.^17^ Thus, when the microgels are treated with in situ metal reduction to introduce nanoparticles and densely packed during jamming, we anticipated that the metal–phenolic networks and large interfacial area would enhance electrical conductivity over hydrogels without the microgel structure (e.g., bulk hydrogels) or where nanoparticles are simply embedded. Further, the intrinsic injectability of granular hydrogels^18^ allows fabrication of 3D printed electroactive patterns (e.g., wearable and flexible electronic devices) and electrophysiological support for biological tissues (e.g., myocardium, skeletal muscles).

**Figure 1:**
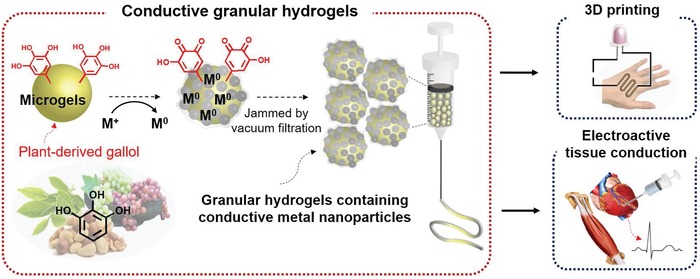
Overall schematic of conductive granular hydrogels inspired by plant‐derived gallol and used for biomedical applications.

To implement this design, chemically modified HA was prepared and characterized. The HA was modified with either methacrylates alone (MeHA), gallol alone (HA‐Ga), or with both methacrylates and gallols (MeHA‐Ga), with ≈36% and ≈13% of disaccharides modified with methacrylates and gallol, respectively (**Figure** **2**a; Figure S1a–c, Supporting Information). Each modification was selected to achieve mechanically stable microgels via methacrylate crosslinking and to improve upon the in situ metal reduction process between MeHA and MeHA‐Ga. For example, MeHA with methacrylate modifications less than 20% formed weak microgels that dissociated during washing and gallol modifications less than 5% showed negligible differences in conductivity when compared to MeHA alone (results not shown). Both MeHA and MeHA‐Ga underwent rapid gelation (e.g., G′ > G″) in the presence of ultraviolet light, whereas HA‐Ga did not (Figure S1d,e, Supporting Information).

**Figure 2:**
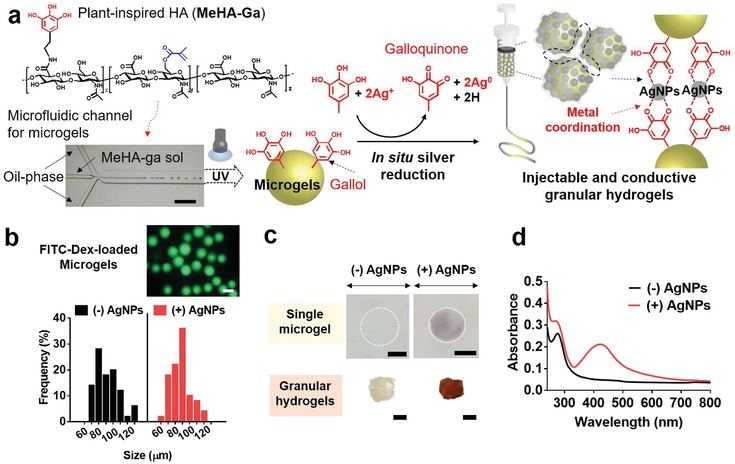
Fabrication of conductive granular hydrogels and their characterization. a) Schematic of the preparation of conductive granular hydrogels using a microfluidic device to form microgels (formed with methacrylated and gallol‐modified hyaluronic acid, MeHA‐Ga) through a water‐in‐oil emulsion, in situ metal reduction by gallol moieties, and then jamming through vacuum filtration. b) Size distribution of microgels without ((−)AgNPs, black) or with ((+)AgNPs, red) AgNPs. The fluorescent image shows the microgels with encapsulated FITC‐Dextran for visualization. Scale bar: 100 µm. c) Optical images of single microgels (top) or granular hydrogels (bottom) either without ((−)AgNPs) or with ((+)AgNPs) in situ metal reduction. The color change is due to the introduction of silver nanoparticles. Scale bar of 50 µm for top images and 3 mm for bottom ones. d) UV–vis spectra of microgel suspensions without ((−)AgNPs, black) or with ((+)AgNPs, red) AgNPs.

The HA microgels were fabricated and subsequently underwent in situ metal reduction to provide electrical conductivity. Microgels of ≈90 µm in diameter were prepared in a microfluidic channel, by generating water‐in‐oil droplets of MeHA or MeHA‐Ga, photocrosslinking with ultraviolet light, and washing from oil (Figure 2a,b). Based on the chemistry used, the microgels will be very stable over time with degradation only in response to hyaluronidase and potentially to hydrolysis on very long time scales. More rapid degradation can be incorporated, such as with protease degradation,[qv: 18b] depending on the application and desired degradation profiles. The gallol moieties in the microgels facilitated in situ silver reduction to introduce silver nanoparticles (AgNPs),^19^ which slightly increased the microgel size and was visualized through a change in color and absorbance of the microgels at 425 nm, demonstrating the quantum plasmon resonance of the AgNPs (Figure 2c,d).^20^ In addition, the silver reduction process was not dependent on pH (Figure S2, Supporting Information). The wavelength in the maximum absorbance (λ_max_) of the microgel solution was slightly blue‐shifted from 425 nm at pH 6 to 414 nm at pH 8, yet the overall width of the absorption peak was similar. The results indicate a broad size distribution of AgNPs in situ generated at both pH 6 and 8 due to the random nucleation of AgNPs. Although methacrylates could in part reduce silver salt ions,^21^ there was only minimal change in microgel color (light yellow) when MeHA was processed into granular hydrogels and the introduction of the gallol groups with MeHA‐Ga resulted in greater electroconductivity than that of MeHA alone (Figure S3, Supporting Information). Thus, the MeHA‐Ga microgels were utilized for subsequent experiments.

The microgels could be jammed into solid materials (i.e., granular hydrogels) and we analyzed rheological properties of the hydrogels. The silver reduction process increased the storage modulus (G′) of the granular hydrogels up to 129.8 ± 15.6 Pa from 25.0 ± 13.2 Pa without reduction (**Figure** **3**a,b). This is likely due to the increased interactions between microgels due to the coordinated network between gallols and the newly generated AgNPs.^22^ Similar to our previous studies,^18^ the granular hydrogels were shear‐thinning and self‐healing, allowing injectability (Figure 3b–e). This was observed through a series of high (1000%) and low (1%) strains, where self‐recovery was observed in granular hydrogels with or without AgNPs across numerous cycles (Figure 3c), as well as qualitatively via ejection from a narrow 27 gauge needle (inner diameter = 0.21 mm) (Figure 3d). Further, the granular hydrogels with AgNPs exhibited shear‐yielding with increased strains (0.1% to 1000%) (Figure 3b) and decreased viscosity with increased shear rates (0.02 to 100 s^−1^) (Figure 3e).

**Figure 3:**
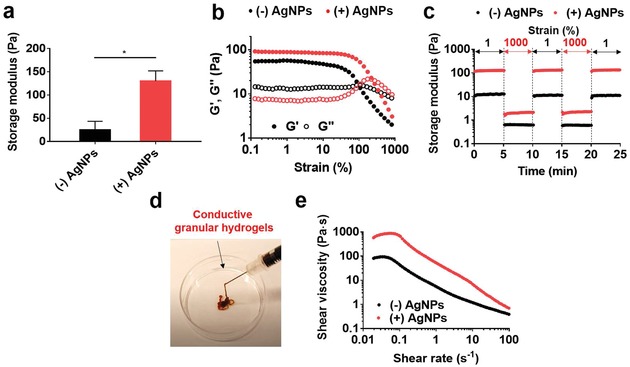
Rheological characterization of granular hydrogels, either without or with AgNPs. a) Storage modulus (G′ at 1 Hz) of the granular hydrogels. Unpaired *t*‐test, *p < 0.05. b) Storage modulus (G′, filled symbol) and loss modulus (G″, open symbol) as a function of strain (0.1–1000%, 1 Hz). c) Evaluation of self‐recovery of the granular hydrogels under alternating strains of 1% and 1000%. d) Macroscopic ejection of the conductive granular hydrogel ((+)AgNPs) from a 27 gauge needle. e) Shear viscosity with increasing shear rates (0.02–100 s^−1^) of granular hydrogels.

Electrical conductivity of the granular hydrogels was also investigated (**Figure** **4**; Figure S4, Supporting Information). It was expected that the electrical conductivity of the granular hydrogels containing in situ synthesized AgNPs would be enhanced due to their large surface area, enabling continuous electrical flow (brown solid line, Figure 4a). Furthermore, the spatial distribution of AgNPs (e.g., inside or surface of the microgels) and their chemical stability in the microgel may also affect conductivity. To explore this, we compared electrical conductivity of four types of hydrogels: bulk hydrogels (no microgel structure) with in situ silver reduction, the granular hydrogels without AgNPs, and granular hydrogels containing AgNPs, either pre‐embedded during microgel fabrication (“pre‐emb”) or in situ synthesized (“in situ”) (Figure 4a).

**Figure 4:**
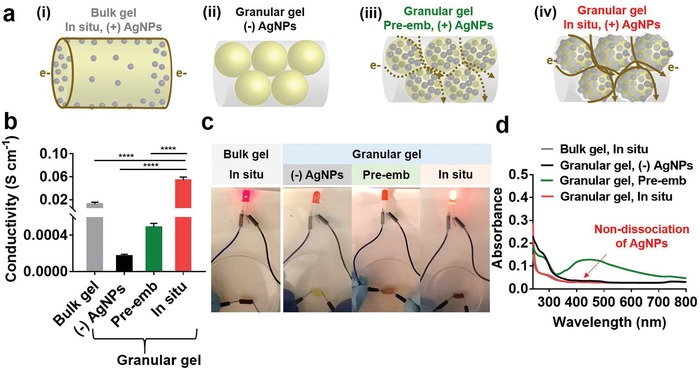
Conductivity of granular hydrogels. a) Schematic of hydrogel structures, including: i) bulk hydrogels with AgNPs through in situ process (“in situ”), ii) granular hydrogels without AgNPs, iii) granular hydrogels with AgNPs pre‐embedded during microgel fabrication (“pre‐emb”), or iv) granular hydrogels with AgNPs through “in situ” process. The brown line describes the proposed electron (e‐) transfer passing through each hydrogel, including a solid line for continuous flow and dashed line for discontinuous flow. b) Electrical conductivity in the various hydrogels. One‐way ANOVA, Dunnett's test for multiple comparisons to “in situ” microgels, ****p < 0.0001. c) LED emitting tests in electrical circuit serially connected with the various hydrogels, showing greatest light intensity for granular hydrogels with microgels treated with the “in situ” process. d) UV–vis spectra after 24 h indicating dissociation of AgNPs from the hydrogels.

The structural differences of the hydrogels affected the electrical conductivity. Limited conductivity was observed in the microgels in the absence of AgNPs (i.e., (−)AgNPs) and the addition of AgNPs improved conductivity across all other groups, at magnitudes dependent on the hydrogel structure and technique to incorporate AgNPs (Figure 4b). For example, the conductivity of the “in situ” microgels (0.05 ± 0.003 S cm^−1^) was five times higher than that of “in situ” bulk hydrogels (0.01 ± 0.003 S cm^−1^), indicating that the hydrogel structure (e.g., granular hydrogel versus bulk hydrogel) is important. The high conductivity of the granular hydrogels is attributed to the large amount of AgNPs present in each microgel, as well as their interfacial packing (Figure S5, Supporting Information). When comparing the amount of silver for the same cross‐sectional area, the Ag in the granular hydrogels was 20.4 ± 1.4 wt%, whereas this was only 6.4 ± 0.9 wt% in the bulk hydrogels (Figure S5a, Supporting Information). In the scanning electron microscope (SEM) images of the bulk hydrogels, AgNPs were observed at random; however, the AgNPs were notably observed in the polymeric layers of the granular hydrogels and displayed a clear peak of “Ag” species in energy‐dispersive X‐ray spectrum (EDS) (Figure S5b, Supporting Information). These results imply that the large interfacial area in the granular hydrogels enhances electrical conductivity, resulting in continuity of electrical flow.

In addition, the morphology and size of microgels may affect the electrical conductivity of granular hydrogels (Figures S6 and S7, Supporting Information). To investigate the influence of the microgel morphology on conductivity, bulk hydrogels were ground into irregular microgels with a broad size distribution of 50–400 µm, jammed after the in situ reduction, and the conductivity measured as above (Figure S6a, Supporting Information). The average conductivity of the ground bulk gel was ≈2.8 fold higher than that of bulk gel, likely due to increased surface area; however, the conductivity was lower than that of the spherical microgels, likely due to the irregularity of the material, which may reduce the surface area particularly with larger particle fragments (Figure S6b, Supporting Information). To investigate the influence of the microgel size on conductivity, granular hydrogels were fabricated from microgels with average diameters of either 90 or 156 µm (Figure S7a, Supporting Information). The storage modulus of the granular hydrogels slightly increased from ≈130 Pa for 90 µm to ≈228 Pa for 156 µm (Figure S7b, Supporting Information); yet, there was only a non‐significant increase in electrical conductivity (Figure S7c, Supporting Information). Thus, microgel size and shape should be considered in the design of conductive granular hydrogels, but the influence on conductivity will likely depend on the magnitude of differences in these parameters.

The spatial distribution of AgNPs is important to control granular hydrogel electrical conductivity. To illustrate this, we prepared controls of granular hydrogels with AgNPs that were pre‐embedded by mixing in the MeHA‐Ga solution before generating microgels (“pre‐emb”). The nanoparticle concentration of 0.6 mg mL^−1^ was selected to match the absorption peak of the AgNPs (A_425_) in the “in situ” microgels, and after jamming there was no difference in the storage moduli of both granular hydrogels with AgNPs (Figure S8, Supporting Information). The “pre‐emb” microgels may sequester free electron transfer, causing low conductivity, which was observed with a 100‐fold higher electrical conductivity of the granular hydrogels from “in situ” microgels when compared to those from the “pre‐emb” microgels (Figure 4b). For qualitative assessment, we also performed the light‐emitting diode (LED) tests to investigate conductivity, where the light intensity was observed in a serially connected circuit with a cylinder of hydrogel (Figure 4c). The bright emission was notably detectable in the “in situ” microgels, whereas weak emission was observed in “in situ” bulk hydrogels and negligible intensity was observed in the “pre‐emb” microgel and “(−)AgNPs” groups. Importantly, these results indicate that electrical flow depends on the method of AgNP incorporation into microgels even though the same amount of AgNPs was present.

In situ synthesized AgNPs also have greater chemical stabilization of AgNPs by gallol moieties via spontaneous metal–phenolic network when compared to physically pre‐embedded AgNPs. This is illustrated through measurement of the AgNPs release from the hydrogels after 24 h (Figure 4d), where the “in situ” synthesized AgNPs were not yet released from the hydrogels, while the physically pre‐embedded AgNPs were rapidly dissociated. To better understand this, attenuated total reflectance infrared (ATR‐IR) spectra of the “in situ” or “pre‐emb” microgels were analyzed (Figure S9, Supporting Information). For the “in situ” microgels, the sharp peaks at 1613 cm^−1^ for C=O and 1315 cm^−1^ for C—O bonds^23^ were shifted to lower wavenumbers of 1579 and 1294 cm^−1^, respectively; however, there was no detectable peak shift in the “pre‐emb” microgels. The peak shift is due to bond formation between silver and oxygen of gallols/oxidized galloquinone coupled with reduction of Ag^+^ to Ag^0^, indicating the steric stabilization of AgNPs synthesized in situ. Taken together, the microgel assembly improves electrical conductivity, and in situ metal reduction coupled with gallol oxidation enables chemically stable, continuous electrical flow, resulting in high conductivity.

For biomedical applications, the injectability of the conductive granular hydrogels is advantageous for 3D printing of an electroconductive pattern, as well as direct electrophysiological bridging of biological tissues. The in vitro cytotoxicity of the granular hydrogels (e.g., “(−)AgNPs” and “in situ, (+)AgNPs”) (Figure S10, Supporting Information) was first assessed by collecting releasates from granular hydrogels and adding to fibroblast cultures. A viability of 82.8 ± 4.3% was measured with most cells staining viable for the conductive hydrogels (Figure S10a, Supporting Information). We next performed extrusion‐based 3D printing to test the filament formation of the conductive hydrogels (**Figure** **5**).^18^, ^24^ Due to the granular structure, these hydrogels were easily extruded to form a smooth filament, which displayed a brown color with a densely packed microgel morphology (Figure 5a). For physical support of a free‐standing printed conductive microgel pattern, a two‐layered lattice was successfully printed on a HA film (Figure 5b). The “(−)AgNPs” microgels or “pre‐emb” formulations were also printable on the film; however, the constructs were not free‐standing and easily disrupted, likely due to the hydrated surfaces of the microgels and no means to stabilize the interface between microgels. In contrast, the silver reduction of AgNP‐coated “in situ” microgels and resulting metal–phenolic stabilization after jamming improve this interface stability and allowed easy transfer of the printed lattice onto porcine myocardium tissue (Figure 5c), demonstrating facile fabrication of the electroconductive pattern and potential application to implantable/wearable devices.

**Figure 5:**
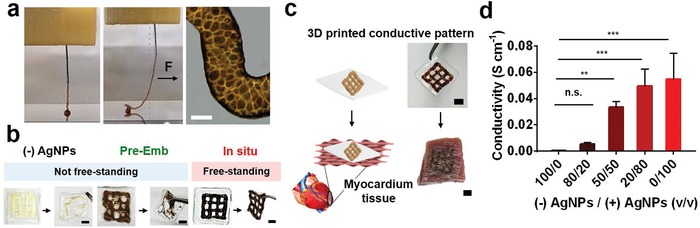
3D printing of conductive hydrogels. a) Images of 3D printing process of the conductive granular hydrogels and morphology of the printed filament. The black arrow indicates a physical force (F) applied to the filament with needle translation during printing, showing self‐supporting of the filament. b) Printability of the granular hydrogels fabricated from microgels without AgNPs (“(−)AgNPs”) or with pre‐embedded (“pre‐Emb”) or in situ synthesized (“in situ”) AgNPs on the polymeric film and their free‐standing stability when removed with forceps. c) Schematic and images of transferring of the printed lattice of the conductive microgels onto porcine myocardium. d) Conductivity of extruded filaments as a function of volumetric mixing ratio v/v of the “(−)AgNPs” or “in situ, (+)AgNPs” microgels. One‐way ANOVA, Dunnett's test for multiple comparisons to “100/0” filament, n.s. for not significant, **p < 0.01, ***p < 0.001. Scale bars: 100 µm for (a) and 3 mm for (b,c).

Depending on the application, it may be important to alter the material conductivity. Although there are numerous ways in which to do this, such as with the gallol concentration or in situ reduction process, we chose to illustrate this through altering the ratio of microgels with and without AgNPs during the jamming process and formation of granular hydrogels (Figure 5d). Extruded filaments (e.g., (−)AgNPs/(+)AgNPs = 0/100) exhibited electroconductivity corresponding to that reported above for cylinders of granular hydrogels (0.05 ± 0.003 S cm^−1^) and the conductivity of filaments was dependent on the volumetric ratio of the microgels with/without AgNPs (e.g., (−)AgNPs/(+)AgNPs displaying the ratio). Specifically, an increase in the ratio of the “(+)AgNPs” microgels enhanced the conductivity of the filament from 0.0005 S cm^−1^ for 0% of the “(+)AgNPs” microgels to 0.05 S cm^−1^ for 100%. The desirable electroconductivity is promising to match a variety of biological tissues, such as myocardium where conductivity ranges from 0.0016 longitudinally to 0.00005 S cm^−1^ transversely.^25^

The conductive granular hydrogels supported electrical conduction as a bridge between biological tissues (**Figure** **6**). To illustrate this, an ex vivo test was performed using two freshly isolated skeletal muscles of the rat hind limb (Figure 6a). The muscle tissues were spaced at a distance of 5 mm apart with/without the granular hydrogels (e.g., “(−)AgNPs,” “in situ,” and “pre‐emb” for (+)AgNPs). In this experiment, stimulating electrodes were placed in one muscle and recording electrodes were placed in the second unstimulated muscle to monitor the electromyography recordings and to record the action potential amplitude. When bridging the two muscles with the granular hydrogel from unmodified microgels (i.e., (−)AgNPs), contraction of the unstimulated muscle was not detectable (Movies S1 and S2, Supporting Information). In contrast, for the “in situ” microgels or “pre‐emb” groups, noticeable contraction of the muscle was observed (Movies S3 and S4, Supporting Information). However, the conductive granular hydrogels from microgels fabricated with the in situ process showed higher action potential amplitude (e.g., 9.85 ± 0.92 mV at 100 mA) when compared to those from the “pre‐emb” microgels (e.g., 3.9 ± 2.03 mV at 100 mA) at all stimulation currents (Figure 6b,c), demonstrating greater conduction ability of the “in situ” microgels.

**Figure 6:**
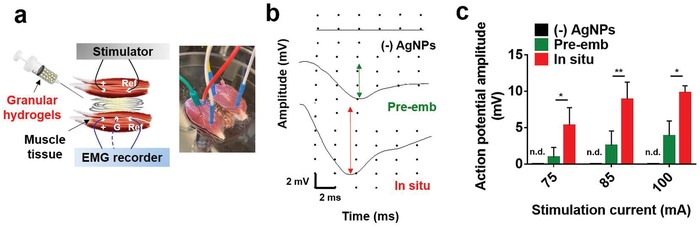
Bridging of conductive tissues with granular hydrogels. a) Schematic and representative image of ex vivo electrical tissue conduction test using isolated skeletal muscles and injection of granular hydrogels between the muscles. b) Electromyogram signals detected in the unstimulated muscle at a stimulation current of 100 mA. The arrows indicate action potential amplitude. c) Action potential amplitude detected in the unstimulated muscle during tissue bridging with the granular hydrogels, from (−)AgNPs, in situ, or pre‐emb microgels. n.d. for not detectable, *p < 0.05, **p < 0.01, two‐way ANOVA.

This work introduces injectable and conductive granular hydrogels that can be explored for numerous applications beyond those shown here. Since the metal–phenolic coordination network involved in microgel interactions is dynamic and reversible, applications should consider the potential for dissociation of the microgels and whether further secondary crosslinking is needed for hydrogel stability. For instance, conductivity decreased from 0.05 to 0.01 S cm^−1^ when incubated for 5 days at body temperature due to a gradual loss of the physicochemical network between microgels (Figure S11, Supporting Information). This may be particularly important with use in vivo and the physicochemical environment should be considered in specific material design. In addition, to apply this system to tissue repair, cell‐encapsulated microgels (e.g., without metal nanoparticles) can be mixed with these conductive granular hydrogels, enhancing cell viability and controlling electrical conductivity. As with all nanomaterials, cytotoxicity and biocompatibility should be addressed for a specific application. Other therapeutics, such as growth factors for tissue regeneration, could also be loaded into the microgels for further biofunctionality. Fortunately, the system that is introduced is highly modular where properties such as mechanics and degradation are readily altered through changes in the macromer design and microgel processing.

In conclusion, we developed an injectable and conductive granular hydrogel via gallol redox chemistry coupled with in situ metal reduction. The granular hydrogels containing in situ synthesized metal nanoparticles exhibited higher conductivity (≈0.05 S cm^−1^) than that of bulk hydrogels and either untreated microgels or microgels with pre‐embedded nanoparticles, due to the large surface area and gallol‐to‐metal coordination. The conductive granular hydrogels allowed 3D‐printable extrusion, fabricating free‐standable constructs on the polymeric film with conductivity as a function of the volumetric ratio of the microgels with/without metal nanoparticles. In addition, the conductive microgels restored electrical conduction by bridging two separated muscle tissues. Our findings present a new technique in the design of soft conductive materials that are also injectable, a promising approach for enhancing electrical conductivity for numerous biomedical applications.

## Experimental Section

*Preparation of Conductive Microgels*: To prepare microgels, mineral oil (Fisher Chemical) supplemented with span 80 (2 w/w%, Sigma‐Aldrich) and MeHA‐Ga (or MeHA) solutions (5 w/v%) with Irgacure 2959 (final concentration of 0.1 w/v%, Sigma‐aldrich) and FITC‐Dextran (1.7 mg mL^−1^, Sigma‐Aldrich, ≈2 MDa) were separately introduced into microfluidic devices. The water‐in‐oil droplets were generated at the junction of four microchannels, two‐side inlets for oil flow (50 µL min^−1^), one center inlet for polymer flow (2 µL min^−1^ for the diameter of ≈90 µm and 10 µL min^−1^ for ≈156 µm), and one outlet for droplets. The droplets were crosslinked under UV irradiation (320–390 nm, 200 W cm^−2^, ≈25 s) and rinsed three times in MilliQ water. For metal reduction, microgels were added to silver nitrate solution (AgNO_3_; 500 × 10^−3^ m, Sigma‐Aldrich) at a volume ratio of 1:6 (microgels: AgNO_3_). After overnight incubation to reduce silver ions, the microgels were finally jammed by vacuum filtration (Steriflip, 0.22 µm pores, Millipore).

*Rheological Analysis*: Rheological properties of the granular hydrogels were measured using AR2000 stress controlled rheometer (TA Instruments) with a 20 mm diameter cone and plate geometry and gap size of 200 µm. The storage (G′) and loss moduli (G″) were measured at room temperature under strain sweeps ranging from 0.1% to 1000% (1 Hz frequency) and frequency sweeps (0.01–10 Hz, at 1% strain). To demonstrate self‐recovery properties, the G′ value of the granular hydrogels was evaluated under cycling strains of 1% and 1000%. Each strain was applied to the samples for 5 min at 1 Hz frequency. The shear‐thinning behavior was also investigated by measuring shear viscosity of the granular hydrogels in continuous flow at shear rates from 0.01 to 100 s^−1^. To demonstrate injectability of the granular hydrogels corresponding to shear‐thinning behavior, a syringe with a 27 G needle (BD syringe) was utilized.

*Electrical Conductivity and LED Emitting Test*: To measure electrical conductivity of each gel (e.g., granular hydrogels without/with AgNPs in situ synthesized or pre‐embedded, bulk hydrogels), the hydrogels with a cylinder shape were prepared in a syringe. Two‐terminal electrical resistance (Ω) was monitored using digital multimeter (Dawson), and the electrical conductivity was calculated as follows

(1) $$\text{Electrical}\;\text{conductivity}\;\left(S\;{\text{cm}}^{-1}\right)\;=\;\frac{1}{R\frac{A}{L}}$$

where *R* is (electrical resistance, Ω), *A* is (cross‐sectional area of the hydrogels (cm^2^) = 3.14 × radius^2^), and *L* is (length of the hydrogels, cm). In addition, each hydrogel was serially connected with a battery and LED, and LED emission was visualized.

*Statistical Analysis*: All statistical analysis was performed using Graphpad Prism 7 software. Data were presented as mean ± standard deviation in triplicate or more. For comparisons of two groups, unpaired *t*‐tests were performed, whereas results of multiple groups were compared using one‐way analysis of variance (ANOVA), post hoc testing (Sidak, Dunnett's comparisons, or Tukey test) with *p* value < 0.05 indicating statistical significance, and two‐way ANOVA.

## Conflict of Interest

The authors declare no conflict of interest.

## Supporting information

Supplementary

Supplementary

Supplementary

Supplementary

Supplementary
