# Supplementary material for: Injectable and Conductive Granular Hydrogels for 3D Printing and Electroactive Tissue Support
Source: Adv Sci (Weinh). 2019 Aug 21;6(20):1901229. doi: 10.1002/advs.201901229 (PMC6794627; doi:10.1002/advs.201901229)
Supplement: Supplementary file 1 — Supplementary [file ADVS-6-1901229-s002.pdf]

Copyright WILEY-VCH Verlag GmbH & Co. KGaA, 69469 Weinheim, Germany, 2019.

ADVANCED SCIENCE
Open Access

Supporting Information

for *Adv. Sci.,* DOI: 10.1002/advs.201901229

Injectable and Conductive Granular Hydrogels for 3D Printing and Electroactive Tissue Support

*Mikyung Shin, Kwang Hoon Song, Justin C. Burrell, D. Kacy Cullen, and Jason A. Burdick**

Copyright WILEY-VCH Verlag GmbH & Co. KGaA, 69469 Weinheim, Germany, 2019.

Supporting Information

Injectable and Conductive Granular Hydrogels for 3D Printing and Electroactive Tissue Support

Mikyung Shin, Kwang Hoon Song, Justin C. Burrell, D. Kacy Cullen, and Jason A. Burdick*

Experimental Section

Synthesis of MeHA and MeHA-Ga: For synthesis of MeHA, sodium hyaluronate (HA; Lifecore Biomedical, 66-99 kDa) was dissolved at a concentration of 1 w/v% in Milli-Q water and reacted with methacrylic anhydride (Sigma-Aldrich, 2.226 mL per 1 g of HA) at pH 8.0–8.5 for 4 hours on ice bath. The solution was then dialyzed in distilled water for 2 days to remove unreacted methacrylic anhydride, using a regenerated cellulose dialysis membrane (molecular weight cutoff 6-8 kDa). The final product was lyophilized.

MeHA-Ga was prepared by N-(3-dimethylaminopropyl)-N'-ethylcarbodiimide hydrochloride (EDC) and N-hydroxysuccinimide (NHS) coupling reaction. Specifically, MeHA (100 mg) was dissolved in Milli-Q water (9 mL) and the pH was adjusted to 4.5 using 1 M HCl. EDC (202 mg) and NHS (243 mg) was sequentially added to the MeHA solution and the pH was adjusted to 5.0. Subsequently, 5-hydroxydopamine hydrochloride solution (136 mg dissolved in 1 mL Milli-Q water, Sigma-Aldrich, 205.64 Da) was added drop-wise to the MeHA/EDC/NHS mixture (pH 4.5) and nitrogen gas was purged for 15 minutes to minimize the oxidation of gallol. The reaction was performed overnight. The solution was dialyzed in acidified distilled water (pH 4.5) containing 100 mM NaCl for 2 days and distilled water alone for 4 hours (molecular weight cutoff 6-8 kDa), and then lyophilized. As a control, HA-Ga was prepared using HA alone in a same method.

The degrees of methacrylate and gallol modifications were determined by proton nuclear magnetic resonance (^{1}H NMR) spectroscopy (DMX 360, Bruker) by dissolving MeHA or MeHA-Ga (8.5 mg) in deuterium oxide (800 μL, Sigma-Aldrich). For evaluation of gallol modification, Folin and Ciocalteu's phenol assay was also performed.[24] The polymers were dissolved in MilliQ water at a concentration of 5 mg mL^{-1}. Each solution (20 μL) was added into a 96-well clear UV-transparent microplate (Corning). Folin and Ciocalteu's phenol reagent (100 μL, 0.2 M, Sigma-Aldrich) was then added to each sample well, followed by the addition of NaOH (80 μL, 1 M). The samples were incubated for 2 hours at room temperature and the absorbance of each sample (A_{750}) was measured using a microplate reader (Tecan Infinite M200). The gallol standard curve (A_{750}) was established over the concentration range from 0.068 to 0.54 mg mL^{-1} with 5-hydroxydopamine solutions.

Microfluidic device: For microfluidic device fabrication, polydimethylsiloxane (PDMS) was poured onto positive molds designed to contain microchannels (MicroFine Green Material, Proto Labs). After overnight incubation at 37 ^{o}C, the device was punched using biopsy punches with 1 mm diameter (Integra Miltex, Kai Medical) inlets and outlets and attached onto PDMS substrates by plasma treatment (Femto Science). In addition, 18 gauge blunt needles (McMaster-Carr) were inserted into the inlet and outlet holes of the device. For water/oil flow through the device, silicon tubing (inner diameter = 0.79 mm, outer diameter =

2.38 mm, Tygon® ABW00001, Saint-Gobain) was used to connect each syringe containing oil or polymer solution.

Microgel characterization: The morphology and diameter of microgels were observed using an epifluorescence microscope (Olympus BX51). For quantification, the diameter of each microgel (e.g., total fifty microgels) was analyzed by ImageJ software. In addition, to demonstrate formation of AgNPs in the microgels, one drop of the granular microgel was dispersed in MilliQ water (200 μL) and the UV-visible spectrum of the solution (100 μL) was measured (Tecan Infinite M200).

Preparation of bulk hydrogels, ground bulk hydrogels, and silver nanoparticle (AgNPs) pre-embedded microgels: Bulk hydrogels with a cylindrical shape were prepared in a syringe with an inner diameter of ~3.9 mm (final hydrogel length = 10 mm). The polymer solution (100 μL, MeHA-Ga (5 w/v%) containing Irgacure 2959 (0.1 w/v%)) was added into the syringe mold and crosslinked under UV irradiation (200 mW cm^{-2}, 10 minutes). After overnight incubation in $AgNO_3$ solution (500 mM), the bulk hydrogels with AgNPs were produced.
In addition, for preparing the ground bulk hydrogels, the MeHA-Ga bulk hydrogels were cut with blades into small hydrogel pieces, and their size was observed using an epifluorescence microscope (Olympus BX51). In the same manner as with conductive granular hydrogels, the hydrogel pieces were added to $AgNO_3$ solution (500 mM) at a volume ratio of 1:6 (hydrogel pieces : $AgNO_3$). After overnight incubation to reduce silver ions, the hydrogel pieces were finally jammed by vacuum filtration.
To prepare AgNPs-embedded microgels, silver nanoparticle ink (Sigma-Aldrich) was mixed with MeHA-Ga solution at a final concentration of 0.6 mg mL^{-1}. In the same manner as for conductive microgels, the MeHA-Ga/AgNPs solution containing Irgacure 2959 (0.1 w/v%) and FITC-Dextran (1 mg) was introduced into the microfluidic device. After crosslinking of the droplets in outlet flow under UV irradiation (320–390 nm, 200 mW cm^{-2}, ≈ 25 s), the AgNPs-embedded microgels were washed three times in MilliQ water, and jammed by vacuum filtration.

SEM and EDS analysis for in situ synthesized AgNPs visualization and quantification: To compare *in situ* synthesized AgNPs amounts in the cross-section of the granular hydrogels or bulk hydrogels, the silver species in the lyophilized hydrogels were analyzed for 120 seconds using EDS equipped with SEM (Hitachi S-4800). The elemental composition (Ag wt% in the hydrogels) were measured for the same reference area of each sample (Area = 40,560 μm^2). In addition, the cross-sectional images of each sample were observed using SEM.

AgNP dissociation profile: To evaluate the dissociation degree of AgNPs from the hydrogels (e.g., granular hydrogels without/with AgNPs *in situ* synthesized or pre-embedded, and bulk hydrogels), Transwell Permeable Supports with 8 μm pores (C3422, Corning) were set to a 24-well plate. The hydrogels (50 μL) were added to each transwell chamber, and fresh MilliQ water (1 mL) was supplemented in bottom wells. After incubation at room temperature, the solution in bottom wells was collected at 24 hours. The UV-visible spectra of the sample solutions (100 μL) were obtained using a microplate reader (Tecan Infinite M200).

ATR-IR study: For chemical analysis of granular hydrogels without AgNPs, with *in situ* synthesized or pre-embedded AgNPs, the hydrogels (50 μL) were lyophilized for one day. The IR spectra of all samples were obtained using a Nicolet 6700 FT-IR spectrometer equipped with an ATR accessory (Thermo Fisher Sci.).

In vitro cytotoxicity: For analysis of cytotoxicity, microgels were washed in MilliQ water, jammed, and used after sterilization by irradiation for 45 min by a germicidal lamp. Releasates from the granular hydrogels (e.g., (-)/(+) AgNPs, *in situ* synthesized) were collected using a transwell chamber insert (Corning® Transwell® polycarbonate membrane with 8 μm pore) in a 24-well plate. The jammed granular hydrogels (50 μL) were ejected onto the transwell membrane and the releasate was collected from the bottom well supplemented with deionized water (1 mL) over 48 hours.
NIH 3T3 fibroblasts were cultured in DMEM supplemented with 10% fetal bovine serum and 1% penicillin/streptomycin, and seeded at a cell number of 25,000 per well in a 24-well plate. After overnight culture, the sample solution (20 μL) was added to each well. The cell viability (%) was analyzed using both Alamar blue assay (Thermo Fisher Sci.) and Live/Dead assay kit (Thermo Fisher Sci.) after one day of culture. For the Alamar blue assay, the cultured cells were washed once using Dulbecco's phosphate-buffered saline (DPBS), culture media (500 μL) was freshly supplemented into each well, and the alamar blue reagent (50 μL) was added. After 2 hours of incubation, the fluorescence intensity of the media (200 μL) was measured using a microplate reader (λ_{exc} = 560 nm, λ_{emi} = 585 nm, Tecan Infinite M200) and cellular viability (%) was calculated as follows.

$$\text{Cell viability (\%)} = \frac{\text{Fluorescence intensty from the cells treated with the eluents}}{\text{Fluorscence intensity from the cells treated with deionized water}} \times 100$$

In addition, for Live/Dead assay, the cells were stained with calcein AM (2 μM)/ethidium homodimer (4 μM) working solution (500 μL) for 1 hour, and observed using an epifluorescence microscope (Olympus BX51).

3D printing of lattice structure and single line: 3D printing of hydrogels was performed using a modified extruder on a commercial 3D Fused Deposition Modeling printer (Revolution XL, Quintessential Universal Building Device).[24] Briefly, the granular hydrogels were added in a syringe with a luer lock needle (21 G for lattice structure, 25 G for single filaments). Two-layer lattices with dimensions of 0.9 cm × 0.9 cm for each layer (or single filaments with length of 1 cm) were fabricated by standard software to generate G-code (Slic3r) via Repetier hardware control. As a substrate, slide glass or an HA film with a dimension of 1.5 cm × 1.5 cm (dehydrated from HA solution (2 w/v%, 700 μL, Lifecore Biomedical, 151-300 kDa)) was used for printing. The printed lattice on the HA film was transferred to sliced piece of porcine heart tissue. The morphology of single line filaments was also observed by an epifluorescence microscope (Olympus BX51). In addition, to demonstrate the desirable conductivity, the granular hydrogels with or without AgNPs were mixed at a volumetric ratio [microgels without AgNPs]/[microgels with AgNPs] of 100/0, 20/80, 50/50, 80/20, and 0/100. The mixture of the granular hydrogels was added in a syringe with 14 G, and extruded to 1 cm of single filament with a cross-sectional diameter of 1.6 mm. Similarly, to measurement the electrical conductivity, two terminal electrical resistance (Ω) was monitored using a digital multimeter (Dawson) and the electrical conductivity was calculated.

Ex vivo muscle experiments: For *ex vivo* tests to demonstrate the conduction ability of the granular hydrogels applied to muscle tissues, two tibialis anterior muscles freshly isolated from Sprague dawley (SD) rats (male) were placed on a petri dish approximately 5 mm apart. For stimulation and electromyogram (EMG) recording, monopolar needle electrodes were placed in each muscle. One muscle included anode and cathode stimulating electrodes, and the other one contained an active electrode located in the bulk muscle, reference electrode in its tendon, and ground electrode in the middle of the active and reference electrode. Prior to applying the granular hydrogels (e.g., (-) AgNPs, (+) AgNPs *in situ* synthesized or pre-embedded) between the two muscles, the primary muscle was stimulated as a control against a petri dish (polystyrene) for use as a baseline. Subsequently, the granular hydrogels were

injected between the muscles, using a 21 G luer lock needle. The primary muscles were stimulated at ~ 75 mA, ~ 85 mA, and ~100 mA, and the electric potential amplitude was recorded using an EMG recorder in the second muscle.

Supporting Figures

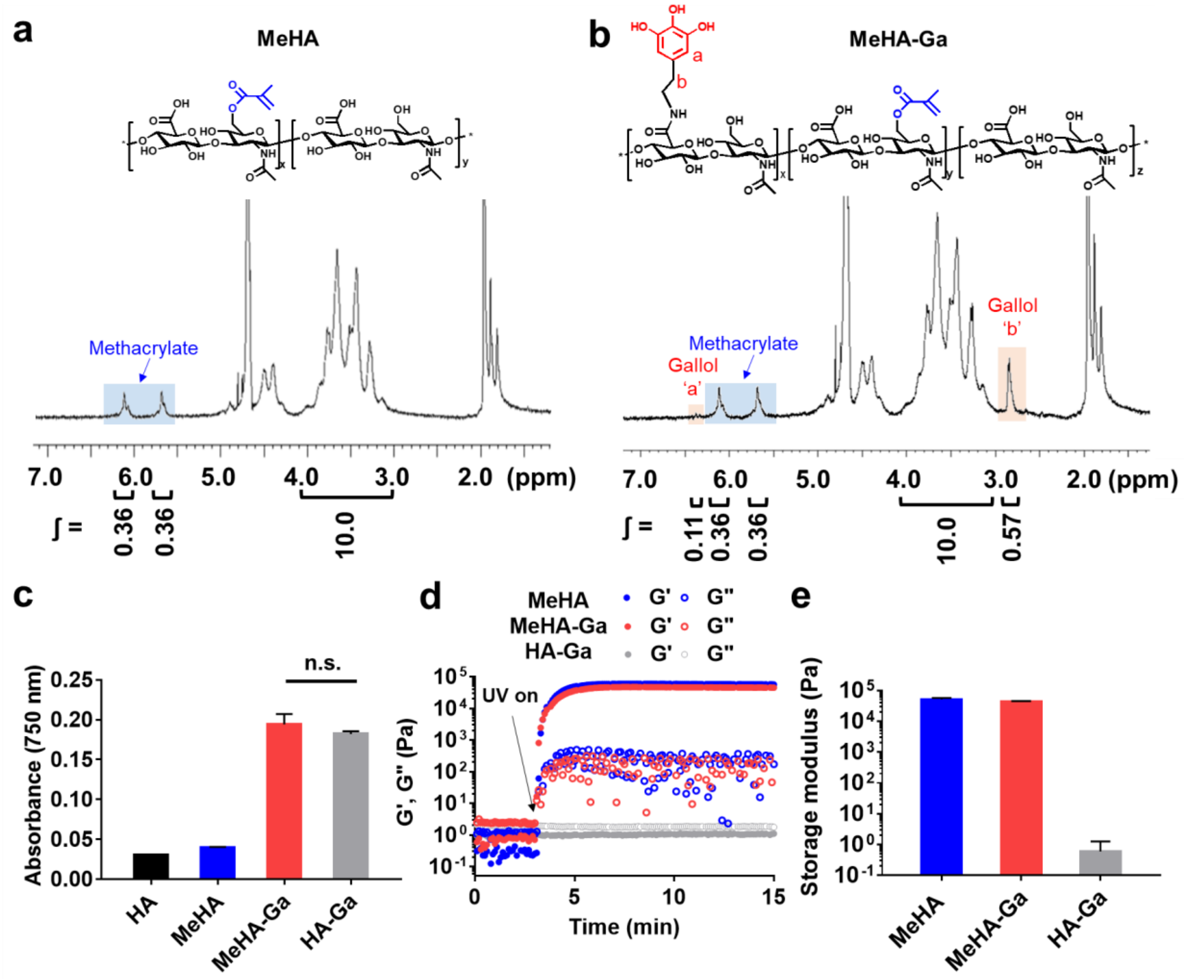

Figure S1. Characterization of methacrylated hyaluronic acid (MeHA) and MeHA-gallol (MeHA-Ga). (a, b) Chemical structure and ^{1}H NMR spectra of synthesized MeHA (a) and MeHA-Ga (b). The degree of modification (~36 % for methacrylate and ~13% for gallol) was determined by integral values (∫) of vinyl protons (δ5.7 and δ6.1) or gallol protons (δ6.4 for 'a' and δ2.9 for 'b' peak), which are displayed in the bottom of the spectra, relative to HA backbone protons (δ3.0-4.1, 10H). (c) Folin and Ciocalteu's phenol assay for HA (black), MeHA (blue), MeHA-Ga (red), and HA-Ga (gray) for quantification of gallol conjugation. One-way ANOVA, Tukey test for multiple comparisons. n.s. for 'not significant'. (d) Photocrosslinking of MeHA (blue), MeHA-Ga (red) and HA-Ga (gray) (10 mW cm^{-2} ultraviolet light). Storage moduli (G') for filled symbols and loss moduli (G") for empty ones. (e) Average storage modulus (G') after photocrosslinking for each polymer at 1 Hz.

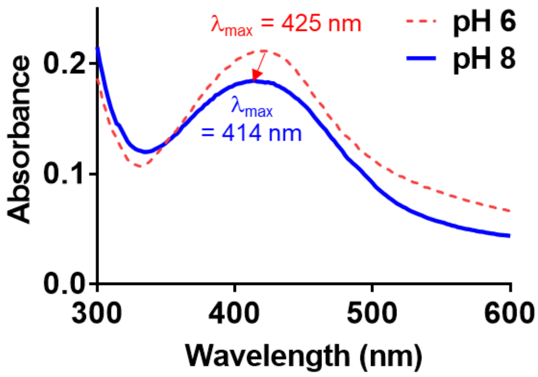

Figure S2. UV-visible spectra of AgNP-containing microgels fabricated by pre-swelling at pH 6 (red) or 8 (blue), followed by the *in situ* Ag reduction process.

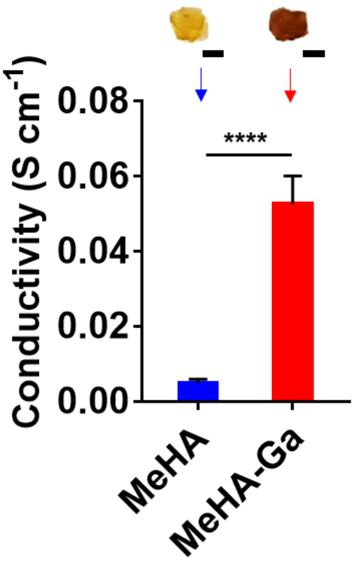

Figure S3. Comparison of electrical conductivity in granular hydrogels fabricated from microgels of MeHA or MeHA-Ga with the reduction process. The inset photo shows the granular MeHA or MeHA-Ga hydrogels. Inset scale bar of 3 mm. Unpaired t-test, ****$p<0.0001$.

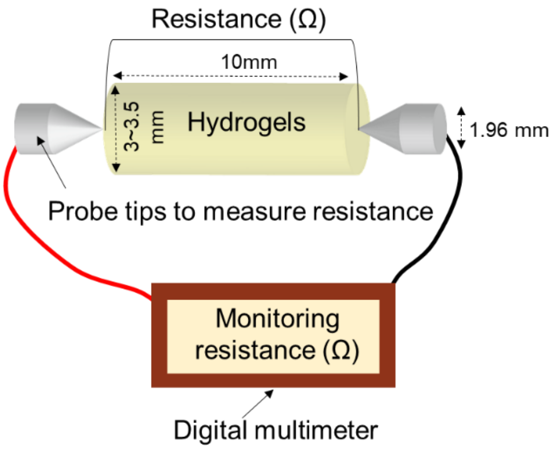

Figure S4. Scheme of experimental setting to measure electrical conductivity of the hydrogels. Digital multimeter, probe tips, and a cylinder type of hydrogels were utilized to measure electrical resistance (Ω).

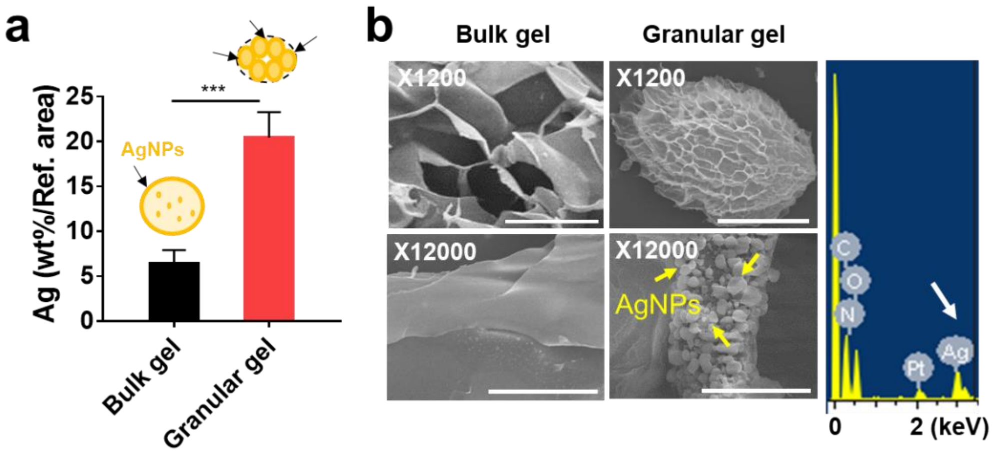

Figure S5. (a) Amount of silver (Ag) present in the cross-sectional area (A = 40,560 μm^2) of bulk hydrogels after the reduction process (black) or granular hydrogels formed form microgels after the reduction process (red). Unpaired t-test, ***P<0.001. The inset scheme shows the expected distribution of AgNPs (e.g., yellow solid line) present in two hydrogels. (b) SEM images (left) of lyophilized bulk hydrogels or granular hydrogels with AgNPs and EDS analysis (right) corresponding to the microgel image. The yellow arrows indicate AgNPs in the microgels and the white arrow shows the detection of 'Ag' species in microgels. Scale bars: 40 μm for top images, 4 μm for bottom images.

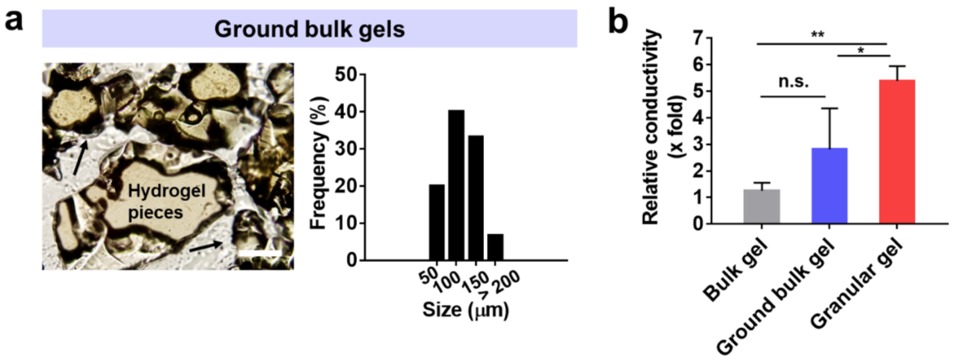

Figure S6. (a) Optical image of ground bulk hydrogel pieces and their size distribution. Scale bar of 200 μm. (b) Relative electrical conductivity of the bulk gel, the granular hydrogel from ground bulk gel microgels, and the granular hydrogel. One-way ANOVA, Tukey test for multiple comparisons. n.s. for 'not significant'. *p < 0.05 and **p < 0.01.

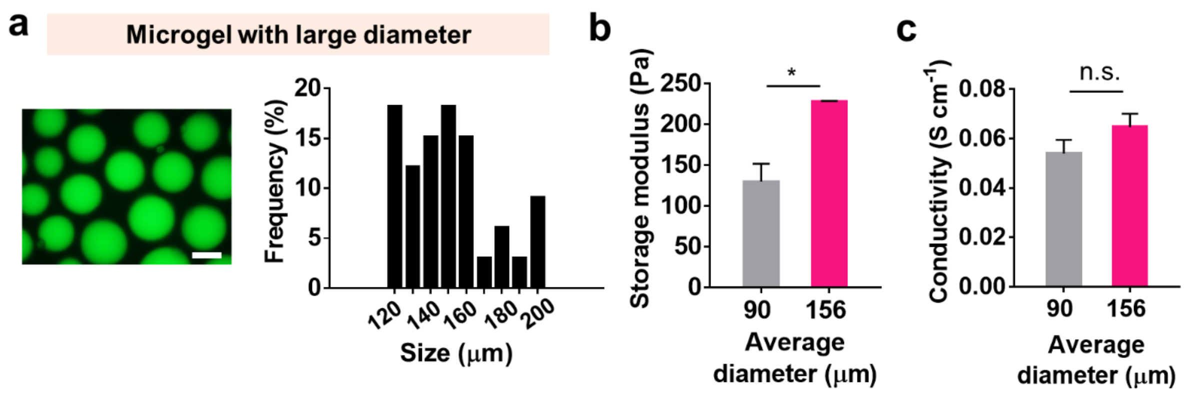

Figure S7. (a) Fluorescent image and size distribution of microgels with an average diameter of 156 μm (encapsulating FITC-Dextran for visualization). Scale bar of 100 μm. (b) Storage modulus (G' at 1 Hz) and (c) electrical conductivity of the granular hydrogels with average diameter of 90 or 156 μm. Unpaired t-test, *p < 0.05, n.s. for 'not significant'.

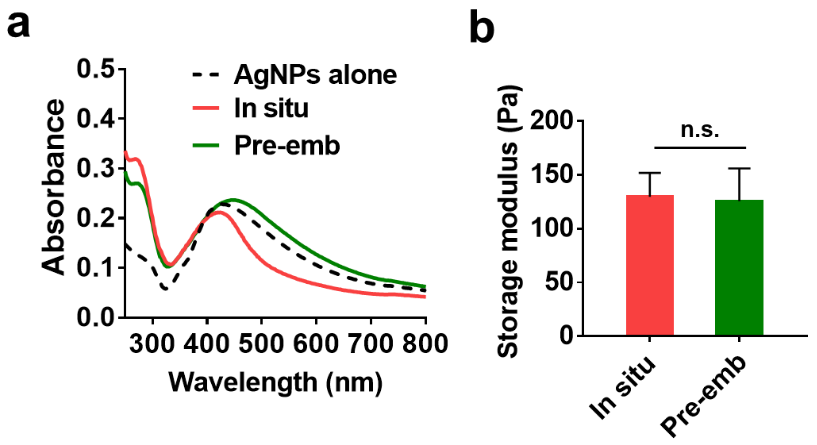

Figure S8. (a) UV-visible spectra of the granular hydrogels with AgNPs either through *in situ* reduction process (red) or pre-embedded (green). The dashed line shows the AgNP solution (without microgel) as a control. (b) Storage moduli of the granular hydrogels with AgNPs either through *in situ* reduction process (red) or pre-embedded (green). Unpaired t-test, n.s. for 'not significant'.

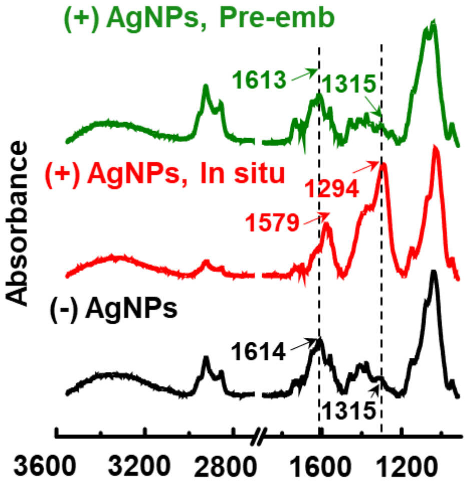

Figure S9. ATR-IR spectra of the granular hydrogels alone (black for '(-) AgNPs') or with AgNPs either through *in situ* reduction process (red) or pre-embedded (green).

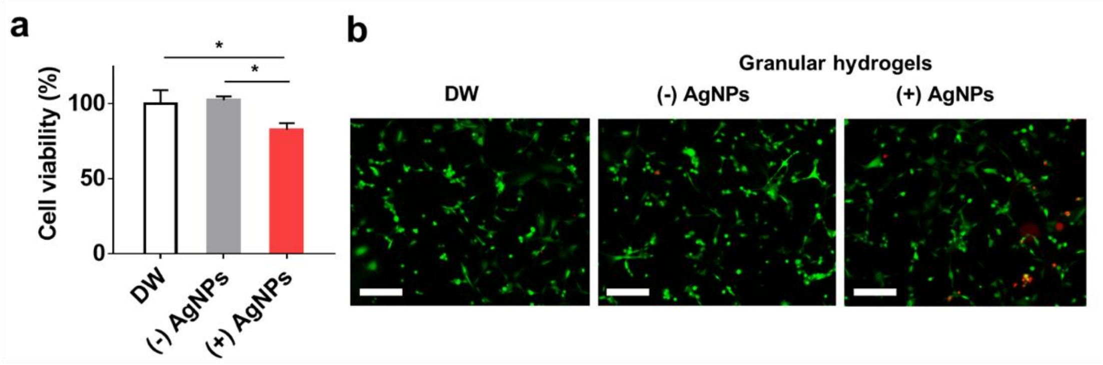

Figure S10. (a) Quantitative cell viability (%) after one day of treatment of the eluents from the granular hydrogels without AgNPs (e.g., (-) AgNPs) or with AgNPs (e.g., In situ, (+) AgNPs) compared to a control treated with deionized water (DW) alone. One-way ANOVA, Tukey test for multiple comparisons. *$p < 0.05$. (b) Fluorescent images of live (green)/dead cells (red) one day post-culture. Scale bar of 100 μm.

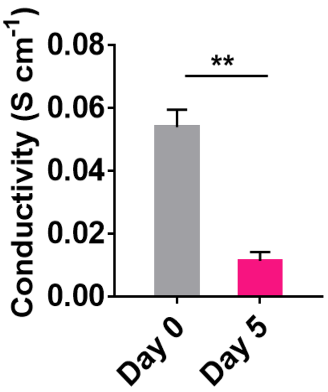

Figure S11. Electrical conductivity of the granular hydrogels for 5-day incubation at body temperature. Unpaired t-test, **$p < 0.01$.
